# TBP Binding-Induced Folding of the Glucocorticoid Receptor AF1 Domain Facilitates Its Interaction with Steroid Receptor Coactivator-1

**DOI:** 10.1371/journal.pone.0021939

**Published:** 2011-07-07

**Authors:** Shagufta H. Khan, Jun Ling, Raj Kumar

**Affiliations:** Department of Basic Sciences, The Commonwealth Medical College, Scranton, Pennsylvania, United States of America; Baylor College of Medicine, United States of America

## Abstract

The precise mechanism by which glucocorticoid receptor (GR) regulates the transcription of its target genes is largely unknown. This is, in part, due to the lack of structural and functional information about GR's N-terminal activation function domain, AF1. Like many steroid hormone receptors (SHRs), the GR AF1 exists in an intrinsically disordered (ID) conformation or an ensemble of conformers that collectively appears to be unstructured. The GR AF1 is known to recruit several coregulatory proteins, including those from the basal transcriptional machinery, e.g., TATA box binding protein (TBP) that forms the basis for the multiprotein transcription initiation complex. However, the precise mechanism of this process is unknown. We have earlier shown that conditional folding of the GR AF1 is the key for its interactions with critical coactivator proteins. We hypothesize that binding of TBP to AF1 results in the structural rearrangement of the ID AF1 domain such that its surfaces become easily accessible for interaction with other coactivators. To test this hypothesis, we determined whether TBP binding-induced structure formation in the GR AF1 facilitates its interaction with steroid receptor coactivator-1 (SRC-1), a critical coactivator that is important for GR-mediated transcriptional activity. Our data show that stoichiometric binding of TBP induces significantly higher helical content at the expense of random coil configuration in the GR AF1. Further, we found that this induced AF1 conformation facilitates its interaction with SRC-1, and subsequent AF1-mediated transcriptional activity. Our results may provide a potential mechanism through which GR and by large other SHRs may regulate the expression of the GR-target genes.

## Introduction

**Figure 1:**
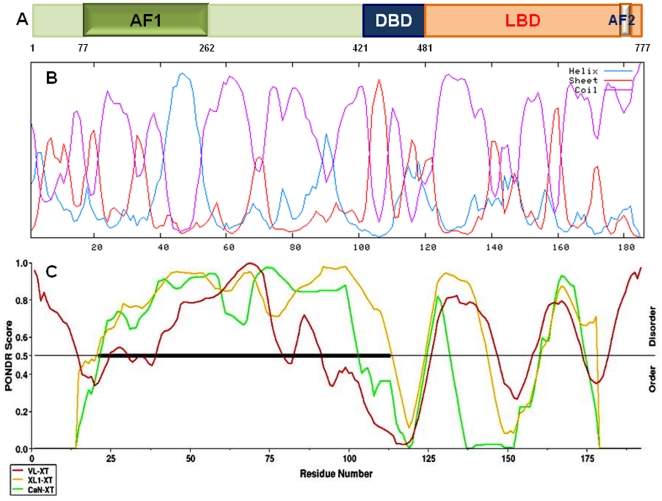
Secondary structural elements predictions of the GR AF1. **A**) Diagram of human GR protein showing major functional domains. **B**) Secondary structural elements predictions of the GR AF1 using HNN method as described [34]. Blue, red, and purple colors indicate helix, β-sheet, and random coil, respectively. **C**) PONDR plot for AF1 protein disorder prediction [35]. X axis shows amino acid numbers in the GR AF1 sequences, and Y axis shows probability score.

Ligand-activated glucocorticoid receptor (GR) regulates transcription of target genes by binding to DNA at specific hormone response elements and by interacting with other coregulatory proteins [1], [2], [3], [4], [5]. Like other members of the steroid hormone receptors (SHRs), the GR possesses a modular structure characterized by three major functional domains: N-terminal domain (NTD), DNA binding domain (DBD), and ligand binding domain (LBD) (Figure 1A). The transactivation activity of SHRs is mainly controlled by two activation function domains, AF1 and AF2 located in the NTD and LBD, respectively [6], [7], [8], [9], [10]. The precise mechanism by which SHRs regulate the transcription of the target genes is largely unknown. This is, in part, due to the lack of structural and functional information about AF1 domain. It has been shown that AF1 is constitutively active and retains 60–80% of the GR transcriptional activity [11], [12], [13], [14]. The AF1 is defined by amino acids 77–262 in the human GR [11], [12], [13], [14]. Due to availability of the LBD crystal structure [15], the relevant structural and functional properties of AF2 have been well characterized whereas it is nebulous in the case of AF1.

In spite of rigorous attempts from several laboratories, we have not yet been able to determine a three-dimensional folded structure of the NTD/AF1 of any member of SHR family. One of the biggest obstacles in knowing the structure of AF1 has been due to its unstructured or intrinsically disordered (ID) conformation in solution, which is found in transactivation domains of several transcription factors (TFs) including SHRs [16], [17], [18], [19], [20], [21], [22]. The GR AF1 recruits other coregulatory proteins, including proteins from the basal transcriptional machinery, e.g. TATA box binding protein (TBP) by creating binding surfaces for these proteins [21], [23], [24], [25]. Other studies have also shown that transactivation domains of several TFs including SHRs undergo a disorder/order transition upon interaction with proteins from the basal transcriptional machinery [26], [27], [28], [29]. We have earlier shown that conditional folding of the GR AF1 is the key for its interactions with its critical coactivator proteins [25].

It is interesting that the ID GR AF1 directly interacts with the TBP, the critical protein that forms the basis for the multiprotein transcription initiation complex. However, the precise mechanism of this process is unknown. *In vitro* transcription studies indicated that the holo-GR acts to stabilize the pre-initiation complex [30], [31], [32]. One possibility may be that TBP binding-induced structured conformation in AF1 is involved in creating a platform for the GR AF1-associated coactivators. In this study we tested whether TBP binding induces structure formation in the GR AF1 such that AF1's interaction with steroid receptor coactivator-1 (SRC-1), a critical coactivator which is important for GR-mediated transcriptional activity, is facilitated. Our results show that TBP binding induced structure formation in the GR AF1 facilitates its interaction with SRC-1, and subsequent AF1-mediated transcriptional activity. Our results provide a potential mechanism through which GR and other SHRs may regulate the expression of the GR-target genes, information essential to understand how specific signals are passed from the receptor to target genes.

## Materials and Methods

### Plasmids

The pGRE_SEAP vector (BD Biosciences, Palo Alto, CA) contains three copies of a GRE consensus sequence in tandem, fused to a TATA-like promoter (*P*_TAL_) upstream from the reporter gene for secreted alkaline phosphatase (SEAP). GR500 encodes amino acids 1–500 of the human GR, plus a five-residue nonspecific extension [32]. TBP was cloned into the pcDNA3.1(+) expression vector (Invitrogen, Carlsbad, CA) and SRC-1 into pEYFP-SRC-1 as described [18]. DNA sequencing was performed on all clones to confirm correct sequences.

### Protein Expression and Purification

The GR AF1 domain was constructed from human GR cDNA digested with *Bgl*II and inserted into an expression vector pGEX-4T-1 (Amersham Pharmacia Biotech) as described [25]. TBP_C_ encoding 181 C-terminal residues of human TBP was expressed in pET-21d vector [24], [33]. The expression and purification of AF1 protein is described [25]. TBP_C_ was expressed in *Escherichia coli* BL21(DE3), and purified on the NTA column (QIAGEN, Valencia, CA) using imidazole step-gradient. Final protein purity of both proteins was greater than 95% as verified by presence of a major single band on SDS-PAGE (Figure S1).

### Surface Plasmon Resonance (SPR) analysis

The kinetics of TBP_c_ binding to GR AF1 was determined by surface plasmon resonance (SPR) on Biacore X-100 plus (GE Healthcare). The binding reaction was carried out at room temperature and in a physiologic buffer (0.01 M HEPES, pH 7.4, 0.15 M NaCl, 50 µM EDTA, 0.05% Tween-20). Purified TBP_c_ was immobilized to Fc2 channel of C1 chip as the ligand at 200–250RU through strong ionic interaction. Fc1 channel was equally treated but without TBP_c_ as the control. Multi-cycle kinetics procedure was employed to measure the binding. AF1 at different concentrations (0.2–6.4 µM) was used as analyte, and sequentially injected over Fc1 and Fc2 channels to measure its binding to TBP_c_. The sensor surface was regenerated by 0.3% SDS after each cycle of binding. The flow rate was kept constant at 30 µl/min. Data from 120 seconds of association and 180 seconds of dissociation were collected. The sensorgrams were normalized by the subtraction of Fc1 from Fc2, and then fitted for kinetics by Biacore X-100 evaluation software using 1∶1 Langmuir binding model (A+B→AB). The affinity (K_D_) was calculated from the equation K_D_ = k_d_/k_a_, where k_a_ is association rate and k_d_ is dissociation rate.

### Circular Dichroism (CD) Spectroscopy

The far-UV CD spectra of the purified recombinant AF1, TBP_C_, and AF1∶TBP_C_ mixtures were recorded at 22°C on a Jasco 815 spectropolarimeter by using a 0.1-cm quartz cell, with a bandwidth of 0.5 nm and a scan step of 0.5 nm. The spectra were recorded at a fixed AF1 protein concentration (4.5 µM) and varying concentrations of TBP_C_. All the spectra recorded were corrected for the contribution of solute concentrations. Each spectrum is a result of five spectra accumulated, averaged, and smoothed.

### Structural predictions for the GR AF1

Network Protein Sequence Analysis was performed using (http://npsa-pbil.ibcp.fr) as described [34]. Predictions for the degree of disordered profile, and Charge/Hydropathy analyses were performed using PONDR prediction (www.pondr.com), as described [35], [36], and Fold Index was performed (http://bip.weizmann.ac.il/fldbin/findex) as described [37]. IUPred analysis was performed (http://iupred.enzim.hu) as described [38].

### Immunoprecipitation Assay

5 µl of antibody for SRC-1, and 50 µl of protein A/G-agarose beads conjugate were incubated for 4 h at 4°C. HeLa nuclear extract containing 0.5 mg of total protein, and 10 µg of purified AF1 and/or TBP_C_ were mixed together and incubated for 2 hr at 4°C in a separate tube, and added to the beads, followed by incubation for another 2 h at 4°C. The beads were centrifuged, washed thoroughly, resuspended in SDS loading buffer, and boiled for 5 min to release bound proteins. The released proteins were resolved by SDS-PAGE and immunoblotted with a GR AF1 antibody after transfer onto a polyvinylidene difluoride membrane as described previously [25]. The results are expressed as means ± the standard error. Levels of significance were evaluated by a two-tailed paired Student *t* test, and a *P* value of <0.05 was considered significant.

### Cell culture and transient transfection

CV-1 cells (American Type Culture Collection) were grown at 37°C in minimal essential medium with Earle's salts (Invitrogen) supplemented with 10% (vol/vol) fetal bovine serum (Atlanta Biologicals, Norcross, GA). Cells were subcultured every 2 to 3 days. CV-1 cells were plated on a 24-well plate (1000 µl/well) one day before the transfection and transfected by using Lipofectamine 2000 (Invitrogen) according to the manufacturer's protocol. Transfected cells were maintained at 37°C in 5% CO_2_ and 95% air for the duration of the experiment. Transfection efficiency was normalized by using immunoblot analysis with specific antibody against AF1.

### Reporter gene assays

We used the SEAP reporter system due to its high signal-to-noise ratio and quantifiable transcriptional activity without the need for cell disruption. CV-1 cells were cotransfected as described above with 0.13 µg of pGRE_SEAP reporter vector; 0.13 µg of pECFP-GR500, pcDNA3.1-TBP, and/or pYFP-SRC-1. The total amount of DNA added was kept fixed by the addition of empty pECFP vector. Medium (25 µl) was collected 24 h later and tested for the presence of SEAP (Great EscAPe SEAP detection kit; BD Biosciences) according to the manufacturer's protocol. The data from different experiments were normalized to GR500 activity. The results are expressed as means ± the standard error. Levels of significance were evaluated by a two-tailed paired Student t test and a *P* value of <0.05 was considered significant.

## Results

### Analyses of ID characteristics of the GR AF1 domain

**Figure 2:**
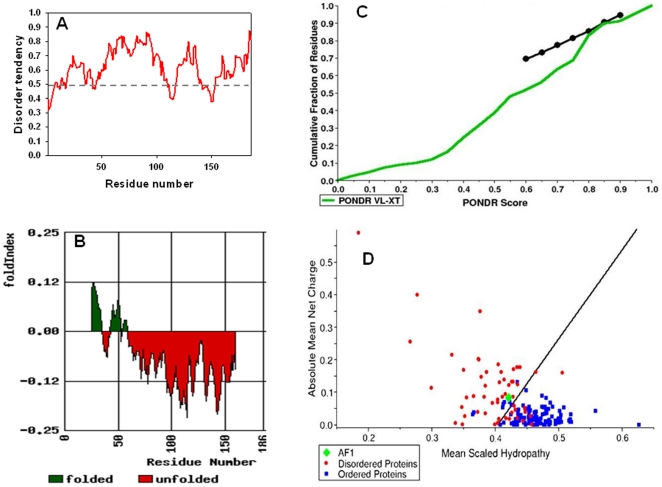
The GR AF1 domain possesses the characteristics of an ID protein. **A**) IUPred plot for AF1 protein disorder prediction [38]. Score above 0.5 are considered to be disordered sequences. **B**) Fold Index showing the probability of AF1 sequences for the propensity to fold [37]. **C**) Cumulative fraction of AF1 residues showing ID PONDR score of AF1 [36]. **D**) Charge-hydropathy analysis using Uversky plot [35], [36]. The plot of the mean hydrophobicity vs. mean net charge of 54 completely disordered proteins (red), and 105 completely ordered proteins (blue). The solid line represents the border between ordered and disordered proteins. The cyan square corresponds to AF1.

We performed secondary structural analysis of the GR AF1 domain (amino acids 77–262) using Network Protein Sequence analysis. It is evident from this analysis that a significantly large number of amino acid sequences represent a random coil configuration (Figure 1B). Further calculations reveal that more than 67% of AF1 sequences contain random coil conformation with only a small proportion as helix and sheet (data not shown). Predictor of Naturally Disordered Regions (PONDR) analysis for ID prediction confirms the ID nature of AF1 (Figure 1C) as evident from the PONDR Score obtained from three different methods (more than 0.5). Similar results were obtained using Prediction of Intrinsically Unstructured Proteins (IUPred) analysis (Figure 2A). To further confirm these findings, we used FoldIndex method of disorder prediction, which predicts the probability of a protein/peptide to fold. A large red area (unfolded) compared to small green area (folded) suggests that a large fraction of the AF1 is unfolded (Figure 2B). Uversky et al. [36] have introduced a method for the analysis to distinguish ordered and disordered protein conformations based only on net charge and hydropathy. We applied this method to the GR AF1 sequences. It is evident from the results that AF1 falls within the ID proteins (Figure 2C). These results were further confirmed from the PONDR Scores obtained as a function of cumulative fraction of residues (Figure 2D) in which the black line plot separates the boundary of database proteins that possess globular structure. It is evident from the green dot plot of AF1 that it falls within the range of ID proteins. Together, data support the notion that AF1 possess characteristics of an ID protein.

### Kinetics analysis of TBPc∶GR AF1 binding by surface plasmon resonance (SPR)

**Figure 3:**
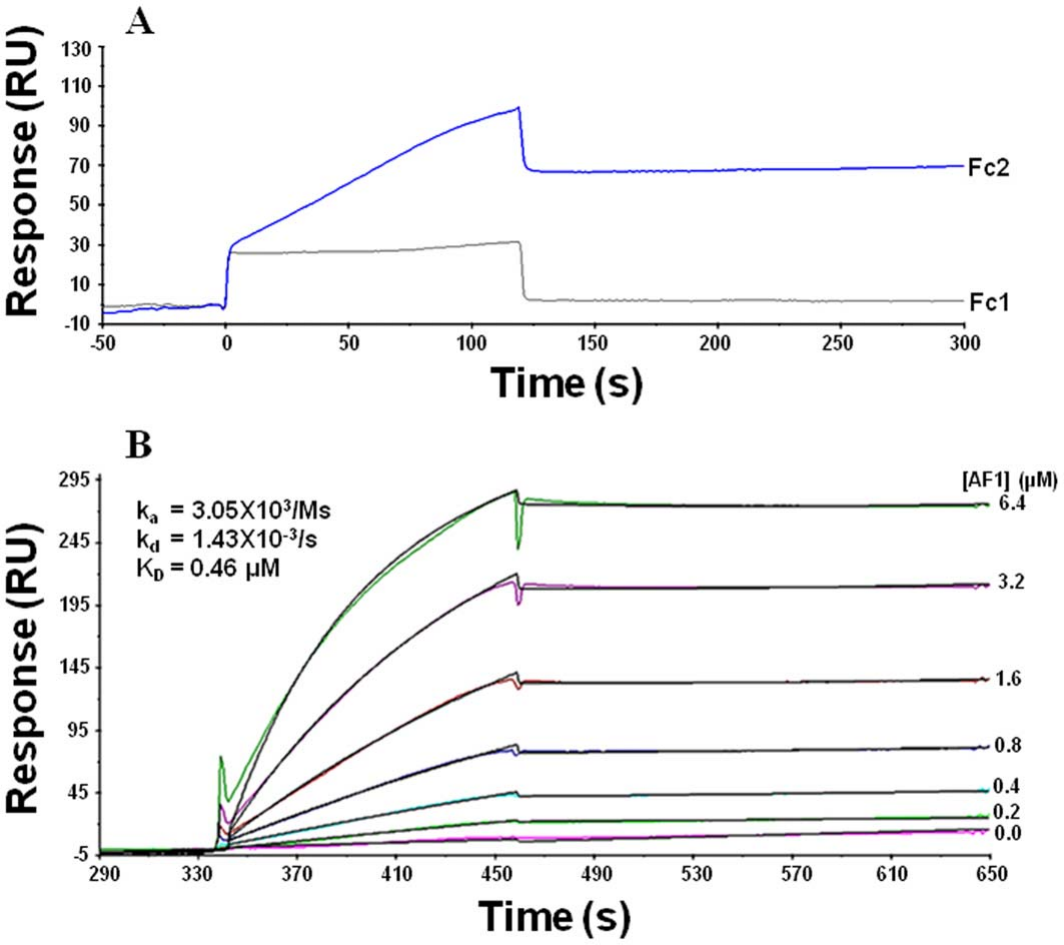
Analysis of the binding of the GR AF1 to TBP by SPR. TBP was immobilized to the Fc2 channel of C1 chip as the ligand, the Fc1 was equally treated but without TBP as the control channel. AF1 was used as the analyte to measure the binding as described in “Materials and methods”. A multi-cycle kinetics assay was run by Biacore X-100 plus. **A**) Raw sensorgrams of Fc1 and Fc2 to show the specific binding of AF1 to TBP. **B**) The adjusted sensorgrams (Fc2-Fc1) were overlaid and fitted for kinetics with 1∶1 binding model. The measured sensorgrams are shown in color, and the fitted ones in black. The experiments were repeated twice.

To measure the binding kinetics of AF1 to TBP by SPR, TBP was immobilized to C1 chip as the ligand through strong ionic interaction, which was based on the high positive charge of TBP_C_ at physiological pH (pI = 10.3). The GR AF1 was used as an analyte. Low density of TBP_C_ (200–250RU) was immobilized to eliminate mass transport limitation and heterogenic ligand for the kinetics assay. The regeneration was optimized to remove both TBP_C_ and AF1 after the binding. Fresh TBP_C_ was immobilized for each cycle; therefore, the activity of ligand was same during the whole process of kinetics assay. As shown in Figure 3A, AF1 exhibited specific binding to TBP_C_ as indicated by the normal sensorgram response in the Fc2 assay channel, whereas the control Fc1 channel was just the perturbation due to the analyte. The sensorgrams were fitted well using 1∶1 binding model (Figure 3B; as shown by black line overlapped with experimental color lines in each case). Meanwhile the dissociation was very slow, implying that there could be an induced conformational change upon the initial binding that caused the formation of stable complex. In fact, we have previously shown that AF1 undergoes order/disorder transition under specific conditions [21], [25].

Since the conformational changes are taking place in AF1, and we used AF1 as the analyte in our SPR assay, it is difficult to be detected by this method. On the other hand, in single cycle SPR kinetic assay, we observed that at lower concentrations, the binding patterns are similar; however, at higher concentrations, the response became weaker and showed different kinetic behavior (data not shown), suggesting that there may be conformational changes occurring in the AF1 when initial AF1∶TBP complex is formed, which is included in the later binding stages. Overall, AF1∶TBP binding displayed a moderate rate of association and slow dissociation with calculated affinity (K_D_) of 0.46 µM, similar to other SHRs, such as binding of estrogen receptor's AF1/NTD to TBP [29]. Comparing the actual SPR response to the immobilized RU of the ligand, we predicted that there could be only one binding site in the AF1∶TBP interaction, which is based on sensogram response theory using equation: R_max_ = R_L_·(MW_A_/MW_L_)·S_m_, where R_max_ is the maximum binding response, R_L_ is the ligand density, MW_A, L_ is the molecular weight of analyte or ligand, and S_m_ is the binding valency; TBP and AF1 have similar molecular weight at 22 kD. Since experimental R_max_ was equal to R_L_, S_m_ should be 1. These results should help us further identify the physical binding sites on AF1 and TBP.

### Binding of TBP induces structure in otherwise ID GR AF1 domain

**Figure 4:**
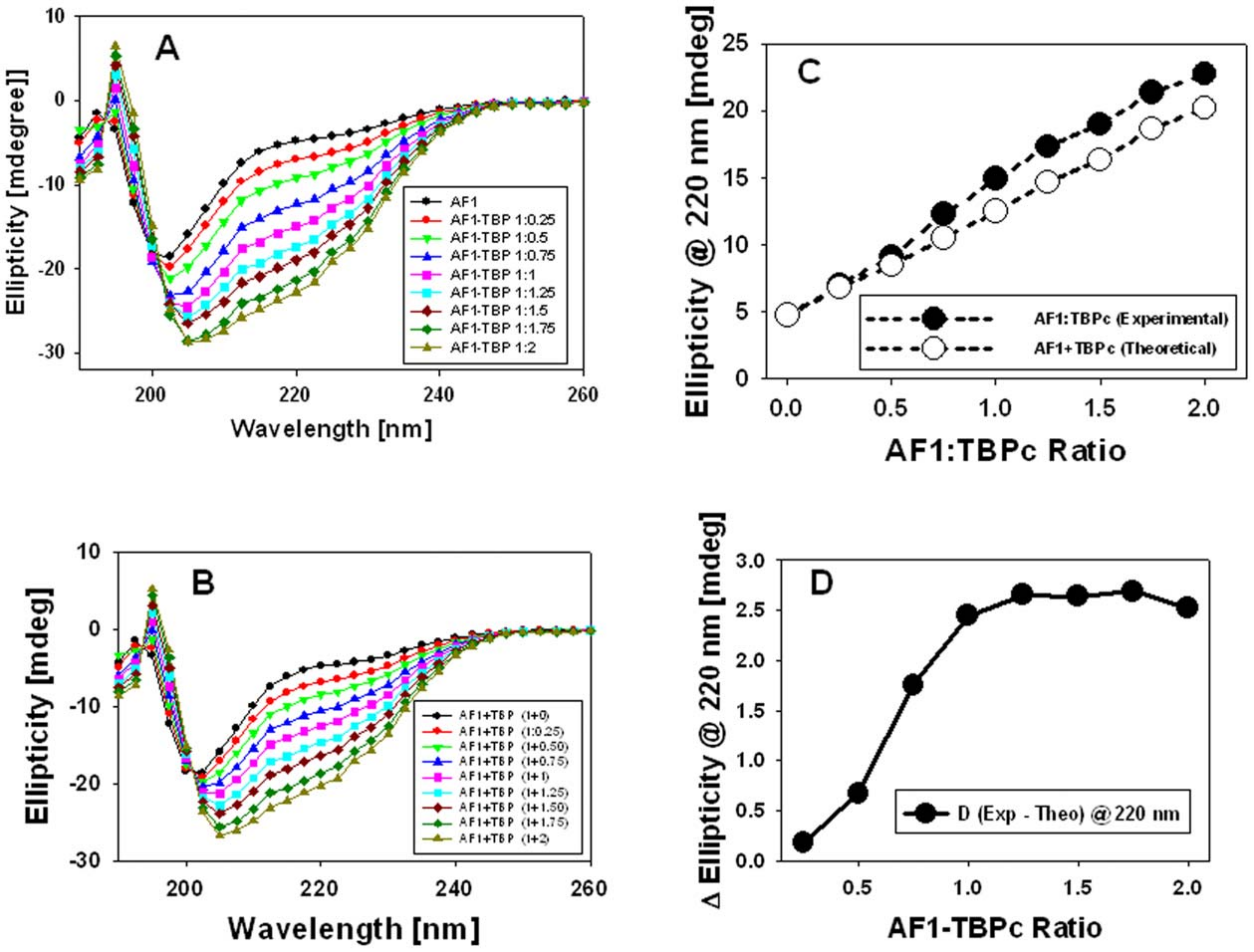
Secondary structural changes in the AF1∶TBP_C_ complex. **A**) Far-UV CD spectra of the GR AF1∶TBP_C_ complex (experimental) at a constant AF1 and varying concentrations of TBP_C_ (as indicated). **B**) Far-UV CD spectra of the GR AF1+TBP_C_ (theoretical sum) at a constant AF1 and varying concentrations of TBP_C_ (as indicated). **C**) A comparison of changes in the ellipticity at 220 nm for experimental and theoretical sums with respect to AF1∶TBP ratio. **D**) TBP-induced conformational transition of AF1 as measured in terms of changes in the ellipticity at 220 nm, and plotted against AF1∶TBP_C_ ratio.

To test the effects of TBP binding on the conformational changes in AF1, we recorded the far-UV CD spectra of purified recombinant AF1, TBP_C_, and a mixture of AF1∶TBP_C_ at 1∶1 ratio. As expected, AF1 alone shows characteristics of an ID protein, and TBP_C_ alone shows that of a globular protein with significant secondary structural elements in it, whereas the complex shows much higher secondary structural elements in comparison to both AF1 and TBP_C_ alone (data not shown). Figure 4A shows the spectra of AF1∶TBP_C_ complex at various ratios ranging from 1∶0.25 to 1∶2. It is clear from these spectra that with increasing concentration of TBP_C_, the complex adopts more secondary structural elements as evident from the increased ellipticity at around 220 nm wavelength followed by a red shift toward 208 nm. When individual data for AF1 and TBP_C_ (at each concentration) are added and plotted (theoretical sum), similar spectra in nature are observed (Figure 4B). When the value of ellipticity at 220 nm was plotted against the increasing concentrations of TBP_C_, a concentration dependent linear relationship was obtained (Figure 4C). A comparison of data from the experimental and theoretical sum suggest that with increasing concentrations of TBP_C_, the complex adopts significantly higher helical content in a concentration dependent manner as evident from the differences in ellipticity at 220 nm (Figure 4C).

**Figure 5:**
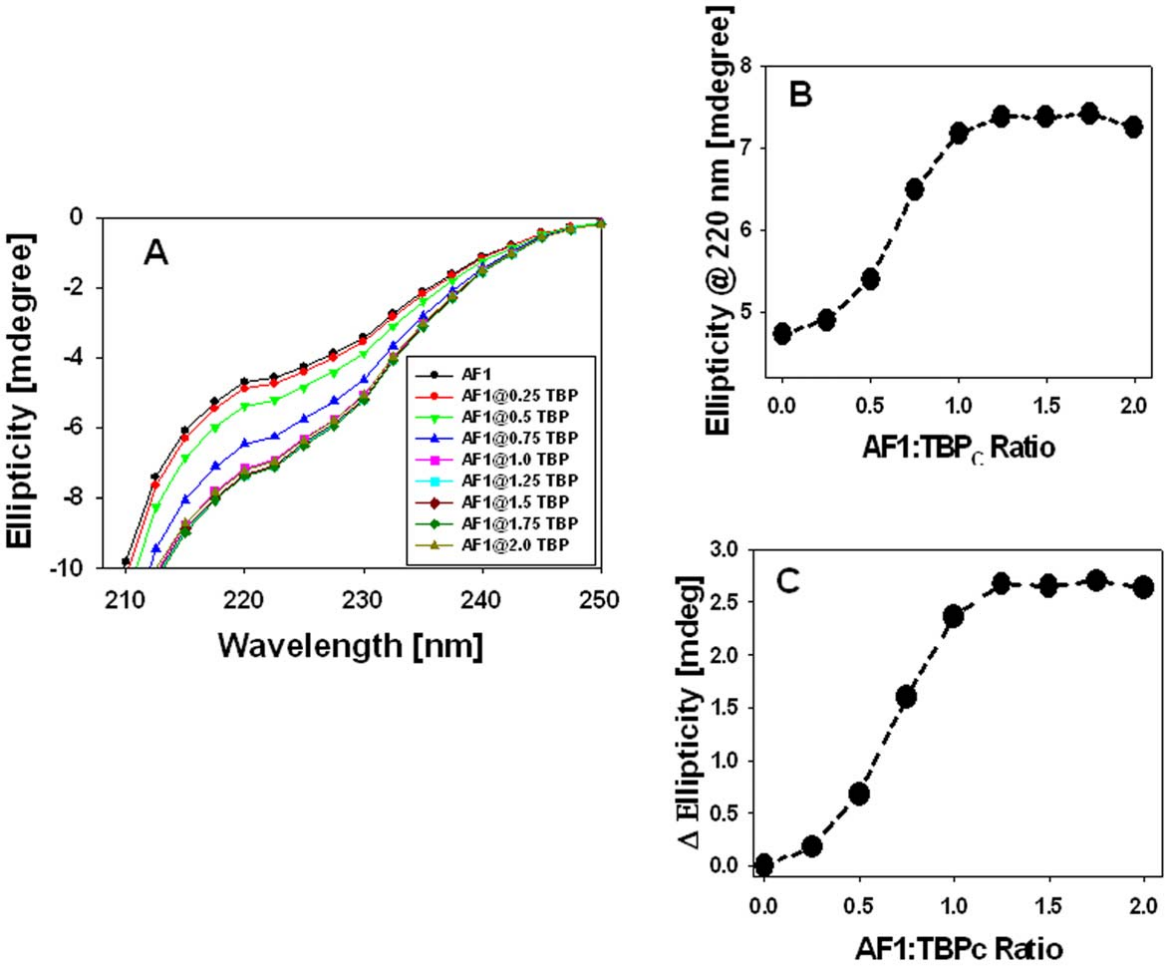
Secondary structural changes in AF1 when complexed with TBP_C_. **A**) Far-UV CD spectra showing AF1 after subtracting the contribution of TBP_C_ in each case. **B**) A plot ellipticity at 220 nm of AF1∶TBP_C_ - TBP_C_ with respect to AF1∶TBP_C_ ratio. **C**) A plot of difference in the ellipticity at 220 nm of AF1∶TBP_C_ - TBP_C_ with respect to AF1∶TBP_C_ ratio.

**Figure 6:**
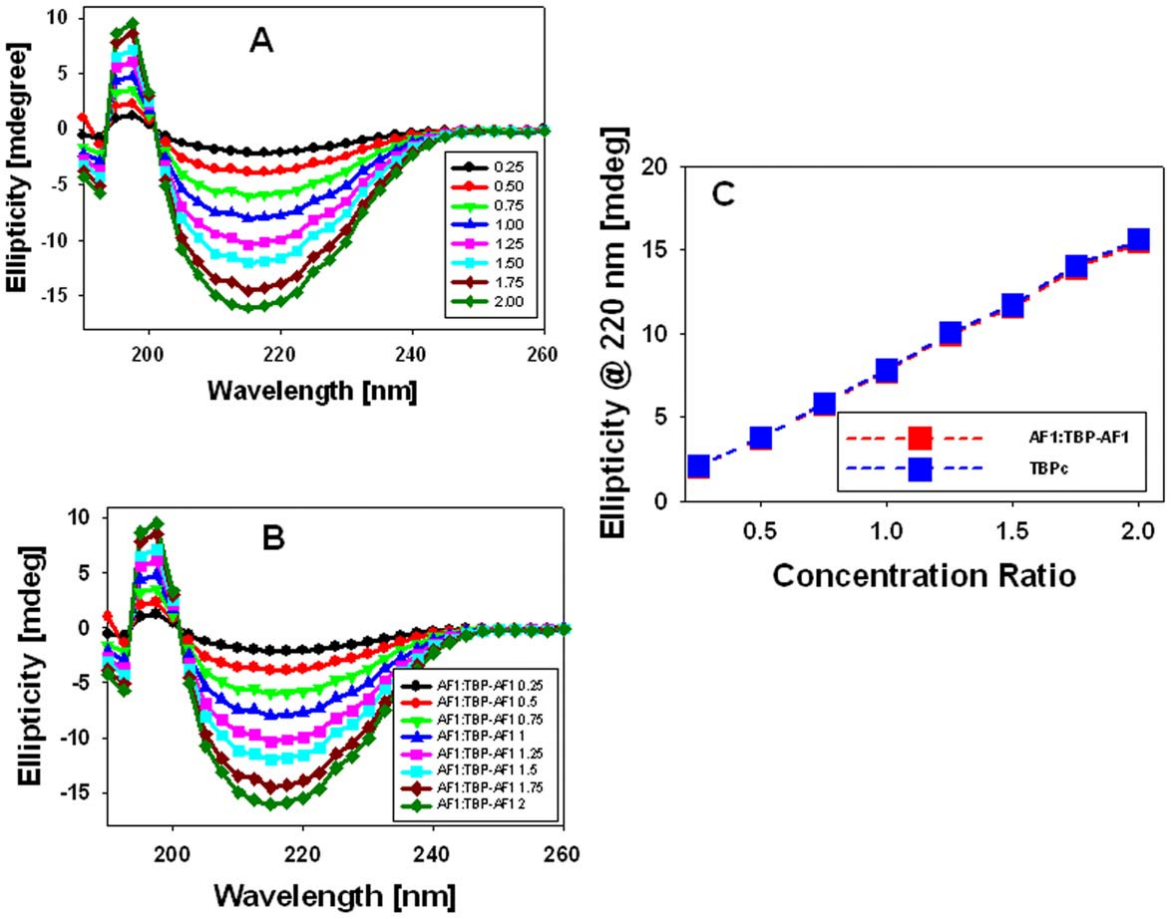
Secondary structural analyses of TBP_C_. **A**) Far-UV CD spectra of the TBP_C_ at various concentrations. **B**) Far-UV CD spectra showing TBP_C_ after subtracting the contribution of AF1 in each case. **C**) A comparison of changes in the ellipticity at 220 nm for TBP_C_ alone and AF1∶TBP_C_ – AF1 at various concentrations of TBP_C_.

To determine the difference between experimental and theoretical sums, we plotted the differences in ellipticity at 220 nm between the experimental and theoretical sum against various concentrations. These results suggest that the helical content in the complex increases up to a ratio of ∼1∶1, and saturates afterword, suggesting that the complex keeps adopting more and more structure formation until the full complex is formed (Figure 4D). To determine whether these structural changes observed in the AF1∶TBP_C_ complex are happening in AF1, TBP_C_, or both, we subtracted the contribution of TBP_C_ from each spectrum and plotted them with respect to AF1 alone (Figure 5A). It is evident from the comparison of the spectra that AF1 adopts significantly higher helical content when complexed with TBP (Figure 5A). Sigmoidal curves shown in Figure 5 B&C represent the absolute changes in the ellipticity at 220 nm and difference in ellipticity in AF1 at each concentration of TBP_C_, respectively. To further determine whether the structural changes observed in the complex are confined to AF1 or TBP_C_ conformation is also changed, we plotted the spectra of TBP_C_ alone (Figure 6A) and after subtracting the contribution of AF1 at each TBP_C_ concentration from AF1∶TBP_C_ complex (Figure 6B). When comparing the changes in the ellipticity at 220 nm (Figure 6C), we observed a linear relationship with increasing concentrations of TBP_C_ (blue line) with no significant deviation in TBP_C_ when present in the complex (red line), suggesting that unlike AF1, there were no significant structural changes in TBP_C_ when complex was formed. Together, these results clearly demonstrate that binding of TBP_C_ to AF1 results in an induced structure formation in otherwise ID AF1 domain without any significant perturbation in TBP_C_ structure.

### TBP binding-induced structure formation in the GR AF1 facilitates its interaction with SRC-1

**Figure 7:**
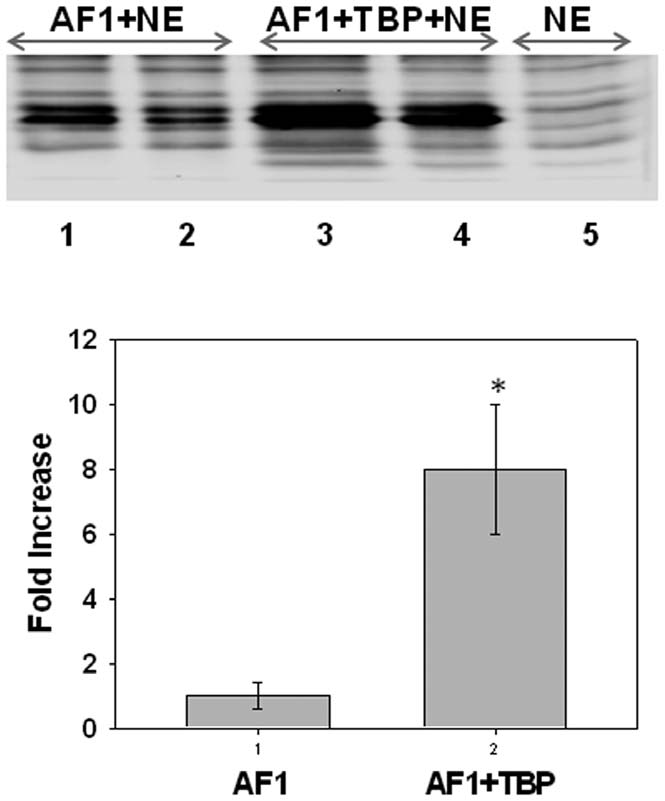
TBP-binding induced conformation in AF1 facilitates AF1's interaction with SRC-1. Immuno-reaction to anti-AF1 antibody after immunoprecipitation (IP) from HeLa nuclear extracts (NE) with SRC-1 antibody (Upper Panel). Lanes 1&2, AF1+NE+beads; Lanes 3&4, AF1+TBP_C_+NE+beads; Lane 5, NE+beads (negative control). An average densitometric analysis of band intensity showing relative fold induction (*p>0.05) (Lower Panel).

It is known that AF1 interacts with SRC-1 to transactivate gene(s), and that conditional folding is important for this interaction [25]. We therefore evaluated whether the conformation induced in ID AF1 domain by TBP binding is important for AF1's interaction with SRC-1. Using immunoprecipitation assay, we tested the interaction between the AF1 and SRC-1 from HeLa nuclear extracts. Separate HeLa nuclear extracts supplemented with purified AF1 ± TBP_C_ protein were prepared. The extracts were then incubated with antibody-linked beads specific to SRC-1. The antibody-linked beads were recovered and washed extensively, and the bound proteins were released and resolved by SDS-PAGE. An antiserum to amino acids 150 to 175 of the GR was then used to identify AF1 on the gels. The results of our immunoprecipitation experiments are shown in Figure 7. The blots shown in the Figure 7 are for AF1 (MW∼22 kD) retained after immunoprecipitation as assessed by AF1 antibody. Consistent with previous reports [25], in the case of AF1 alone, we detected a very weak interaction with SRC-1 (Figure 7; Lanes 1 & 2; Upper Panel) from HeLa nuclear extracts. This interaction was significantly increased (Figure 7; Lanes 3 & 4; Upper Panel) when AF1 was bound to TBP_C_, suggesting that TBP binding-induced formation in AF1 facilitates its interaction with SRC-1 (Figure 7). A quantitative analysis of this interaction shows ∼8 fold increase in the bound SRC-1, when AF1 is complexed with TBP_C_ compared to AF1 alone (Figure 7; Lower Panel). These results suggest that TBP-induced conformation in AF1 is important for AF1's interaction with a critical coactivator.

### Effect of TBP and SRC-1 interactions on AF1-driven transcription

**Figure 8:**
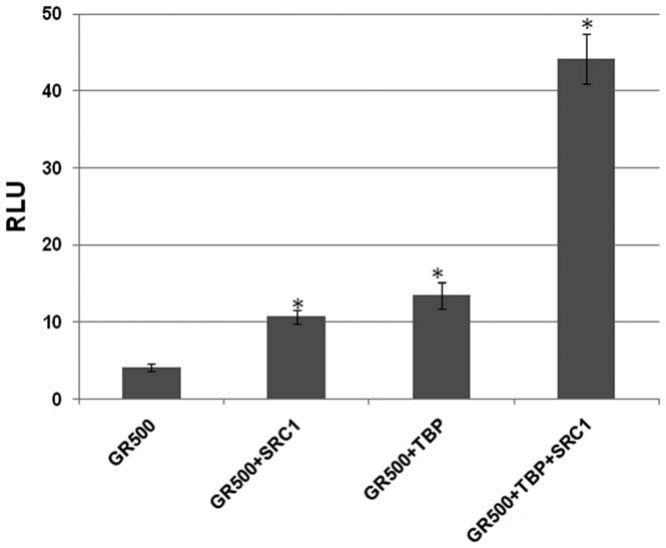
AF1-dependent GR-mediated transcription activation of a promoter containing 3×GRE. CV-1 cells constitutively expressing AF1 in a two domain GR fragment containing entire N-terminal and DNA-binding domains (GR500) were cotransfected with DNA of the pGRE_SEAP plasmid alone or plus DNA for TBP and/or SRC-1. (*p>0.05).

We tested the effects of TBP-induced binding/folding events on AF1-driven transcription using GR-responsive promoters in transient transfection-based reporter assays in GR-deficient CV-1 cells. To test the effects of these coregulators on transcription driven by the human GR AF1, we cotransfected CV-1 cells with a GRE-dependent reporter gene and a constant amount of GR500 expression vector alone or with added vectors expressing TBP and/or SRC-1. The GR500 construct is constitutively active as a transcription factor, while avoiding the possibility of any contribution from AF2 [18]. Lacking the LBD, GR500 is transcriptionally active without steroid and can induce genes and/or apoptosis in cells to nearly the same extent as steroid-bound holo-GR [18], [24]. As expected, GR500 alone significantly increased reporter activity compared to empty vector alone (Figure 8), and input of the plasmids expressing TBP or SRC-1 gene, enhanced the GR500 induction of the GRE-SEAP reporter by ∼2–3 fold (Figure 8). When cells were co-transfected with GR500, TBP, and SRC-1 together, the reporter activity was further enhanced by ∼8 fold. These results strongly suggest that the enhancement of GR-induced transcription by TBP or SRC-1 is achieved through the AF1 region and that TBP binding plays an important role in it by inducing more helical structure in the otherwise ID AF1 region, confirming that TBP-induced structure formation in ID AF1 region aids in facilitating protein-protein interactions between AF1 and coactivators, which subsequently helps drive GRE-mediated AF1 transcriptional activity.

## Discussion

The GR mediates most of the biological effects of glucocorticoids at the level of gene regulation. To regulate the expression of target genes, the GR interacts with several coregulatory proteins including coactivators and corepressors [3]. In recent years, based on the kinetic behavior of the SHR in cells, it has been concluded that the SHRs function very dynamically such that it rapidly and reversibly interacts with its coregulatory proteins, and chromatin and DNA [39], [40]. Requirement of various constellations of coregulatory proteins to regulate GR target genes suggests that the kinetics of these interactions must be variable, depending upon the local concentration and/or binding affinities of these coregulators [3], [41], [42], [43], [44], [45], [46], [47]. Thus, the overall picture is one of a complex, dynamic network controlled by the GR as it interacts reversibly with a variety of other coregulatory proteins. Since many of these cell-specific interactions between the GR and other coregulatory proteins take place through AF1 domain, it is logical to build upon the idea that ID nature of AF1 may be a dominant factor in regulating these events. Of course, for full transcriptional activity, AF1 and AF2 must work synergistically through cross communication, and this is where the flexible structural characteristics of AF1 may play a major role by facilitating protein∶protein interactions.

There are reports showing the evidence for a two-step binding model in which the ID activation domain of c-Myc and estrogen receptor bind rapidly to TBP due to polar interactions and subsequently folds to an ordered conformation [26], [29]. Similar mechanisms have been proposed for the GR as well [21], [24]. TBP has a central role in the basal transcription machinery, and it is known to bind to several TFs, suggesting that the multiprotein complexes involving basal transcription machinery proteins and TF may represent a mechanism through recruitment of TBP to the TATA box. It is generally believed that activation domains of many TFs work through an induced binding/folding mechanism, i.e., they may not be structured until they have recruited and bound their proper binding partners. In this study we show that complex formation between the GR AF1 and TBP is accompanied by a change in protein conformation. An approximate dissociation constant in the µM range for the interaction between AF1and TBP was obtained. However, we were not able to make a complete kinetic analysis of the interaction due to certain technical limitations. A slower dissociation suggests that once the complex is formed, AF1 must have undergone structural rearrangement such that AF1 has now adopted a more stable folded conformation. This is consistent with our findings of increased helical contents in AF1 when complexed with TBP.

Thus, the emerging picture is that ID transactivation regions become folded in concert with target factor interaction, and TBP seems to be a major coregulatory binding partner protein in this process. The question, therefore, arise whether there could be a unified mechanism of TBP binding/folding events on the action of ID activation domains of TFs. We have earlier shown that TBP interacts with the GR AF1 in cells [24]. We have also shown that conditional folding of AF1 is critical for its interaction with SRC-1 [18]. Our present studies certainly support the idea. However, the clear picture will emerge only when the 3-D structures of these complexes are available. Unlike AF2, no single interaction motif has been identified for AF1 coregulatory proteins, and it appears that ID conformation of AF1 helps in promoting molecular recognition by providing surfaces capable of binding specific target molecules [17], [19], [26], [27], [28], [29], [48], [49], [50], [51], [52], [53], [54], [55], [56]. These AF1 surfaces can be achieved through AF1's interaction with specific target molecules, and our data support that TBP may be one such target molecule, since, unlike several other coregulatory proteins including SRC-1 (which bind to both AF1 and AF2 regions), TBP binds and regulates GR activity mainly through AF1 domain [24], [57]. We propose that the ID nature of the GR AF1 allows it to rapidly “sample” its environment until coregulatory proteins of appropriate concentration and affinity are found [5].

Our results show that TBP binding induces secondary/tertiary structure formation in the GR AF1. This TBP binding-induced folding of the GR AF1 is quite striking in the sense that TBP is the major component of basal transcription machinery, and a cross communication of the receptor with the basal transcription machinery is an essential requirement of regulation of GR target genes. Our identification of SRC-1, a critical coactivator of GR activity is a testimony of this fact. We have earlier reported that the GR AF1-TBP interaction occurs under *in vivo* conditions, and amino acid residues 187–242 of the human GR AF1 and amino acid residues 159–339 of human TBP are critical for this interaction [24]. It is also interesting to note that unlike AF2, activation domain-2 (AD2) and possibly AD1 regions of SRC-1 are involved in its interaction with AF1 [58]. SHRs function in an extremely dynamic situation such that they have the capacity to rapidly form and reform multiprotein complexes involving coactivators/corepressors and/or proteins from the fundamental initiation complex. Thus, TBP binding-induced AF1 conformation may provide platform(s) for inclusion and/or exclusion of specific coregulators, which may dictate the final outcome responsible for the regulation of target genes. These effects of course may be cell- and promoter- specific.

It is a well accepted fact that generally, though perhaps not universally [49], [59] under physiological conditions proteins must have specific structure to carry out their proper functions. In the context of the GR AF1, it could be hypothesized that the GR AF1 domain may be structured *in vivo*, at least when directly involved in transcriptional activation. Our studies support this notion, that when carrying out its transcription-regulating function, AF1 shifts to more structured conformers through specific protein∶protein interaction. Conformational uniqueness of most proteins determines their biological function, and we propose that the ID nature of the GR AF1 can explain much about the GR's observed dynamic behaviors in cells. The ID AF1 region can be thought of as a large collection of rapidly inter-convertible conformers, which can select among available coregulatory proteins to form the basis for building large transcription-regulating complexes on specific promoters. Such complexes can dissociate and re-associate with differing composition.

Our results provide a potential mechanism through which GR AF1 may regulate the expression of the GR-target genes, information essential to understand how specific signals are passed from the receptor to target genes. Because TBP is known to bind to several transcription factors including SHRs through their ID activation domain, our results from this study may provide a mechanism through which ID activation domains form assembly of critical coregulatory proteins and subsequent transcriptional activity. Of course, these effects can further be influenced by other factors such inter-domain interactions, and small molecule ligands and other protein∶protein interactions.

## Supporting Information

Figure S1 **Coomassie-stained SDS-PAGE gel showing purified recombinant AF1 and TBP_C_ proteins.** AF1 consists of amino acid residues 77–262 of the human GR, and TBP_C_ represents amino acid residues 159–339 of the human TBP. MW = Molecular Weight Markers. The numbers on the left show the size of MW markers.
(TIF)
