# Supplementary figures and images for: TBP Binding-Induced Folding of the Glucocorticoid Receptor AF1 Domain Facilitates Its Interaction with Steroid Receptor Coactivator-1
Source: PLoS One. 2011 Jul 7;6(7):e21939. doi: 10.1371/journal.pone.0021939 (PMC3131385; doi:10.1371/journal.pone.0021939)

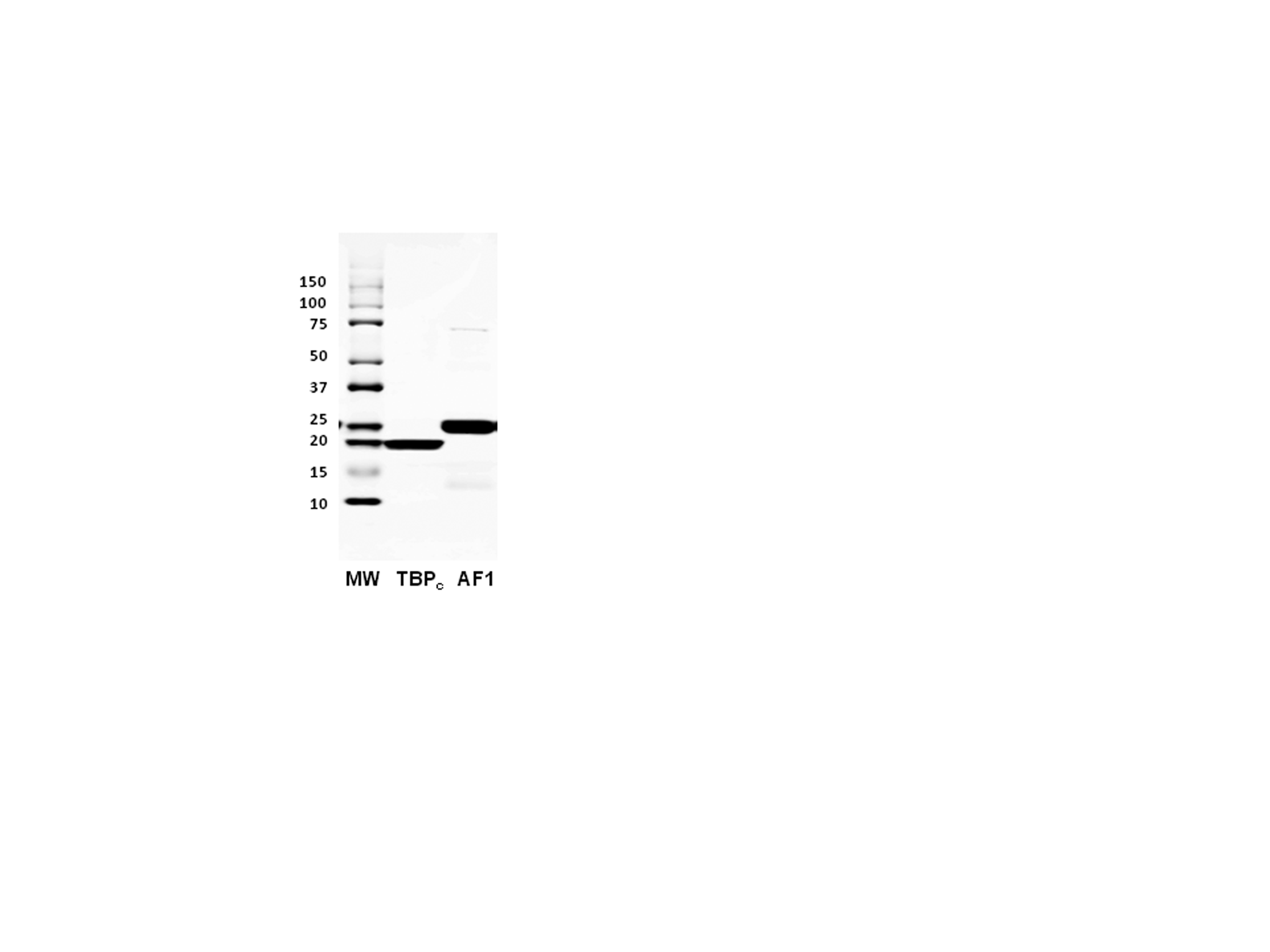

Supplement: Figure S1 — Coomassie-stained SDS-PAGE gel showing purified recombinant AF1 and TBPC proteins. AF1 consists of amino acid residues 77–262 of the human GR, and TBPC represents amino acid residues 159–339 of the human TBP. MW = Molecular Weight Markers. The numbers on the left show the size of MW markers. (TIF) [file pone.0021939.s001.tif]
